# MYCOPLASMA IST3 Results and Antimicrobial Susceptibility in PCR-Positive Urine Samples for *Ureaplasma* spp.

**DOI:** 10.3390/antibiotics15030285

**Published:** 2026-03-11

**Authors:** Rukiye Berkem, Tuğçe Özyol Atkaya

**Affiliations:** Department of Microbiology, Ankara Training and Research Hospital, University of Health Sciences, Hacettepe Mh., Ulucanlar Cd., No: 89, Ankara 06230, Türkiye; rukiyeberkem@gmail.com

**Keywords:** *Mycoplasma hominis*, *Ureaplasma urealyticum*, *Ureaplasma parvum*, antimicrobial resistance, antimicrobial susceptibility testing, urinary tract infections, PCR, MYCOPLASMA IST3

## Abstract

Background: *Ureaplasma* spp. and *Mycoplasma hominis* are urogenital pathogens that may be missed by routine culture, particularly in patients with genitourinary symptoms in whom conventional methods fail to identify an etiologic agent. Limited routine implementation of targeted diagnostics and antimicrobial susceptibility testing (AST) for these organisms may contribute to diagnostic uncertainty and treatment failure. Methods: Seventy-five midstream urine samples submitted for suspected urinary tract infection and positive for *Ureaplasma* spp. according to a q-PCR urinary panel (Bioeksen, İstanbul, Türkiye) were tested the same day with MYCOPLASMA IST3 (bioMérieux, Marcy-l’Étoile, France) to assess growth and antimicrobial susceptibility. Results: q-PCR detected *U. parvum* in 54/75 (72%), *U. urealyticum* in 15/75 (20%), and both species in 6/75 (8%); *M. hominis* was not included in the PCR panel. MYCOPLASMA IST3 showed growth in 70/75 samples (positive percent agreement, 93.33%), while 5/75 (discordance, 6.66%) showed no growth. Among culture-positive samples, 57/70 (81.42%) yielded *Ureaplasma* spp. alone, and 13/70 (18.58%) yielded *Ureaplasma* spp. together with *M. hominis*. Resistance to levofloxacin and tetracycline was observed in 15.7% and 12.9% of Ureaplasma spp. isolates, respectively; resistance to moxifloxacin, erythromycin, and telithromycin was observed in 2.9% of isolates for each agent. In *M. hominis* isolates, no resistance to levofloxacin, moxifloxacin, or tetracycline was observed, whereas clindamycin resistance was observed in 7.7% of isolates. Conclusions: In addition to intrinsic resistance, acquired antimicrobial resistance in *Ureaplasma* and *Mycoplasma* species appears to be increasing; therefore, treatment decisions should be guided by AST whenever feasible. Clinical laboratories should implement appropriate diagnostic methods for these organisms and perform susceptibility testing when indicated to support clinical decision making and optimize antimicrobial selection.

## 1. Introduction

*Ureaplasma (U.) parvum*, *Ureaplasma (U.) urealyticum*, and *Mycoplasma (M.) hominis* are members of the *Mycoplasmataceae* family within the *Mollicutes* class and are commonly referred to as genital mycoplasmas because they can cause urogenital diseases [1,2].

*Mycoplasma hominis*, *Ureaplasma parvum*, and *Ureaplasma urealyticum* may colonize the urogenital tract of healthy adults after the onset of sexual activity and should be considered in patients with urogenital symptoms and pyuria in whom no pathogen is identified by standard culture methods. In routine clinical laboratories, diagnostic testing and antimicrobial susceptibility testing (AST) for these organisms are performed infrequently, which may hinder definitive diagnosis, promote empiric antibiotic use, and contribute to increasing antimicrobial resistance [3,4,5].

Genital mycoplasmas may remain as commensal colonizers; however, they can also cause a range of diseases, primarily affecting the urogenital tract. The most frequently associated urogenital conditions include non-gonococcal urethritis, pelvic inflammatory disease, prostatitis, epididymitis, infertility, endometritis, upper and lower urinary tract infections, overactive bladder, and interstitial cystitis/bladder pain syndrome. In addition, these organisms may contribute to birth-related complications and can cause various infections in neonates born to colonized mothers [1,2,4,5,6].

In patients presenting with urinary tract infection (UTI), *U. parvum*, *U. urealyticum*, and *M. hominis* are not detectable by routine microbiological culture methods. Because these organisms are often not specifically tested for, current knowledge regarding their prevalence and clinical impact remains limited [3,6,7].

Although colonization rates of up to 80% have been reported in women, the available evidence remains limited, and further studies are needed. In symptomatic women, treatment should be guided by AST, and these organisms should be considered in women with chronic urinary tract symptoms [1,2,3,5].

All patients with chronic, unexplained UTI-like symptoms should be evaluated for mycoplasma and ureaplasma infection before proceeding to invasive diagnostic procedures or prolonged treatment, and management should be tailored according to test results. Determining whether a laboratory-confirmed organism represents true pathogenic infection and selecting appropriate therapy ultimately remain the clinician’s responsibility [2,3,8].

Several limitations affect the laboratory diagnosis of these microorganisms. Conventional culture methods require specialized media, have long turnaround times, show limited sensitivity, and often provide identification only at the genus level; therefore, their routine use is restricted. Consequently, faster and more practical diagnostic approaches have been developed, including commercial culture-based assays and polymerase chain reaction (PCR) methods. Commercial liquid culture systems indicate growth based on color change and provide semi-quantitative growth estimates, as well as susceptibility results according to the antimicrobials included in the test. Real-time PCR (q-PCR) is increasingly preferred because of its high sensitivity and specificity, ability to differentiate species, rapid turnaround time, and potential to detect antimicrobial resistance [1,2,7,9,10,11,12].

Because mycoplasmas lack a cell wall, β-lactam antibiotics are ineffective, and they do not synthesize folic acid, trimethoprim and sulfonamides are not used for treatment. The limited antimicrobial susceptibility profile of *Ureaplasma* and *Mycoplasma* spp. further complicates therapy. Tetracyclines and fluoroquinolones are commonly preferred; however, resistance associated with TetM- and ParC-mediated mechanisms has increasingly been reported [6,13,14]. Antimicrobial resistance varies according to regional antibiotic use patterns; therefore, knowledge of local resistance profiles is essential to guide appropriate therapy and support effective resistance management [4,15].

The European Committee on Antimicrobial Susceptibility Testing (EUCAST) does not provide standardized susceptibility testing methods or clinical breakpoints for these organisms. Although the broth microdilution and agar dilution methods recommended by the Clinical and Laboratory Standards Institute (CLSI) are considered reference approaches, they are labor-intensive and difficult to implement routinely. Commercial microdilution assays are easier to perform; however, careful attention is required to ensure that the antimicrobial minimum inhibitory concentrations (MICs) included in these tests and the interpretation criteria are appropriate and consistent with the stated breakpoints [2,14].

In this study, we aimed to evaluate growth and AST results obtained with MYCOPLASMA IST3 in midstream urine samples in which *U. parvum* and/or *U. urealyticum* were detected by q-PCR and to compare key methodological characteristics of the two approaches.

## 2. Results

Of the 75 patients included, 44% were from the obstetrics and gynecology clinic, 28% from urology, 26.67% from internal medicine, and 1.33% from pediatrics; among the 33 patients referred from obstetrics and gynecology, 11 (33.33%) were pregnant (Table 1).

Presumptive clinical diagnoses were urinary tract infection in 86.67% of patients and other genitourinary infection-related conditions in 13.33% (Table 2).

**Figure 1:**
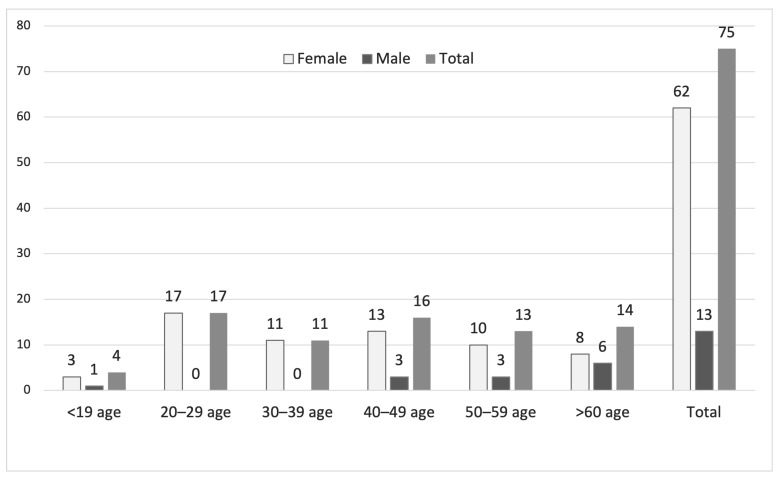
Age and sex distribution of patients with q-PCR-confirmed *Ureaplasma* spp. positivity.

Among the 75 patients included who were q-PCR-positive for *Ureaplasma* spp., 62 (83%) were female, and 13 (17%) were male. The highest number of female patients was in the 20–29-year age group (*n* = 17), whereas the highest number of male patients was in the ≥60-year age group (*n* = 6). Notably, although 28 women were in the 20–39-year age range, no men were observed in this age group. Of the 75 *Ureaplasma* spp.-positive samples, *U. parvum* was detected in 54 (72%), *U. urealyticum* in 15 (20%), and both species in 6 (8%). Among the 62 female patients, *U. parvum* only was identified in 46 (74.2%), *U. urealyticum* only in 10 (16.12%), and both species in 6 (9.68%). Of the 13 male patients, 12 were older than 40 years; *U. parvum* was detected in 8 (61.53%) and *U. urealyticum* in 5 (38.47%). In contrast to female patients, co-detection of *U. parvum* and *U. urealyticum* was not observed in males. Groups were not matched or balanced a priori; therefore, differences in sex distribution across species categories reflect the underlying referral population (Figure 1).

Among the 75 samples in which *Ureaplasma* spp. was detected by q-PCR, growth was observed in the MYCOPLASMA IST3 assay in 70 (positive percent agreement, 93.33%), whereas no growth was observed in 5 samples (discordance, 6.66%). Among the 70 culture-positive samples, 57 (81.42%) yielded *Ureaplasma* spp. Alone, and 13 (18.58%) yielded concomitant growth of *Ureaplasma* spp. and *M. hominis*. No sample showed *M. hominis* growth alone.

**Figure 2:**
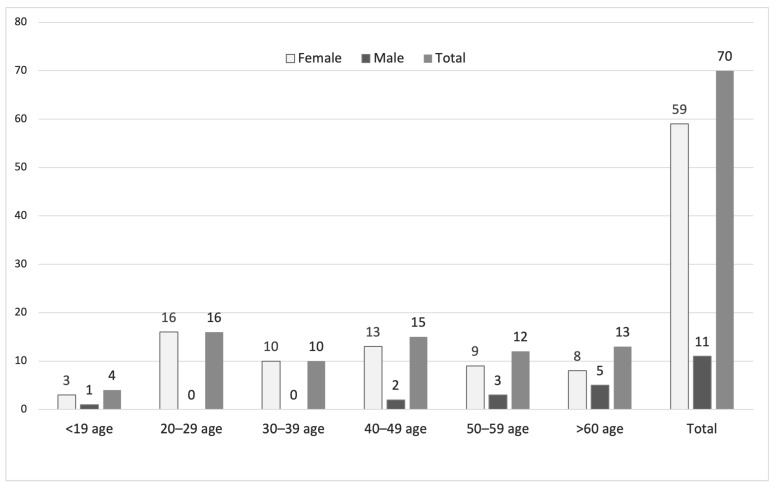
Age and sex distribution of patients with *Ureaplasma* spp. growth detected by MYCOPLASMA IST3.

Among the 70 patients with *Ureaplasma* spp. growth, 59 (84.29%) were female, and 11 (15.71%) were male. Most female patients (*n* = 39) were in the 20–49-year age group, whereas most male patients (*n* = 10) were older than 40 years; in contrast to females, no male patients were observed in the 20–39-year age group. The five samples that were q-PCR-positive for *Ureaplasma* spp. but showed no growth in the MYCOPLASMA IST3 assay belonged to three female patients (aged 29, 37, and 51 years) and two male patients (aged 42 and 60 years) (Figure 2).

**Figure 3:**
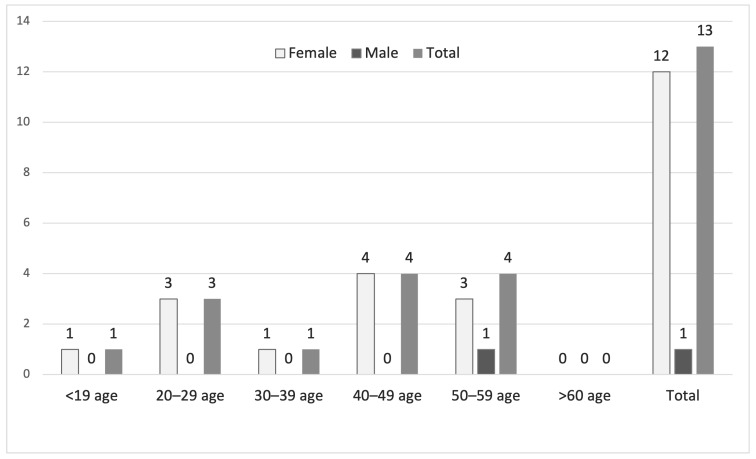
Age and sex distribution of patients with *M. hominis* growth detected by MYCOPLASMA IST3.

Among the 13 patients with *M. hominis* growth, 12 (92.3%) were female, and 1 (7.7%) was male. Most female patients (*n* = 8) were in the 20–49-year age group, and the single male patient was in the 50–59-year age group. Unlike *Ureaplasma* spp., no *M. hominis* growth was observed in either females or males aged ≥60 years (Figure 3).

Among the 75 q-PCR-positive samples, species distribution showed *U. parvum* in 54 (72%), *U. urealyticum* in 15 (20%), and co-detection of *U. parvum* and *U. urealyticum* in 6 (8%). Of the 54 samples identified as *U. parvum* by q-PCR, five showed no in the MYCOPLASMA IST3 assay.

Among the 70 samples with growth detected by the MYCOPLASMA IST3 assay, 57 (81.42%) yielded *Ureaplasma* spp. Alone, and 13 (18.58%) yielded concomitant growth of *Ureaplasma* spp. and *M. hominis*. Of the 70 samples with *Ureaplasma* spp. growth, 64 showed growth at ≥10^3^ CFU/mL, whereas 6 showed growth at <10^3^ CFU/mL. Among the 13 samples with *M. hominis* growth, 9 showed growth at ≥10^4^ CFU/mL and 4 at <10^4^ CFU/mL. The semi-quantitative growth results obtained with the MYCOPLASMA IST3 assay are summarized in Table 3.

**Figure 4:**
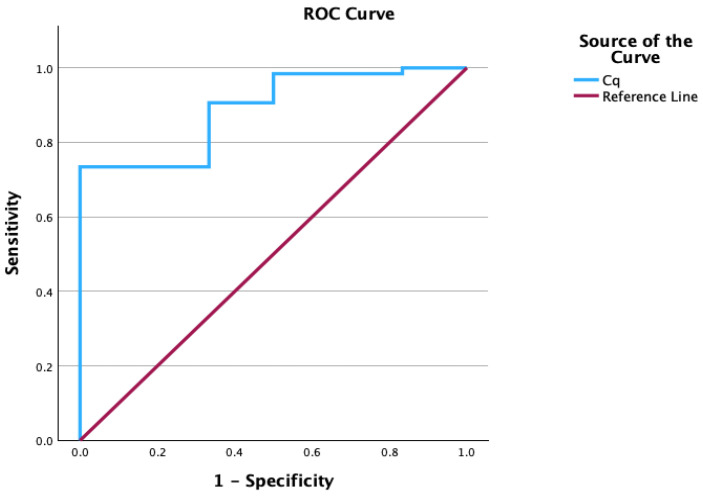
ROC curve for Cq values in detecting ≥10^3^ CFU/mL *Ureaplasma* spp. growth.

The Cq cutoff value was determined in samples with ≥10^3^ CFU/mL *Ureaplasma* spp. growth detected by the MYCOPLASMA IST3 assay. Accordingly, a Cq threshold of 20.17 yielded the highest sensitivity and specificity. For samples with Cq values below 20.17, the sensitivity and specificity for detecting ≥10^3^ CFU/mL *Ureaplasma* spp. growth were 73.40% and 66.70%, respectively. The area under the ROC curve was 0.891, indicating good discriminatory performance of the Cq variable. The corresponding 95% confidence interval was 0.785–0.996. The AUC was statistically significant (*p* < 0.001). The ROC curve is presented in Figure 4, and the corresponding values are summarized in Table 4.

The MIC values (µg/mL) and susceptibility categories obtained with IST3 for the 49 samples positive for *U.parvum*, the 15 samples positive for *U. urealyticum*, and the six samples positive for both *U. urealyticum* and *U. parvum* by q-PCR are presented in Table 5.

Among the 70 samples with *Ureaplasma* spp. growth, resistance to levofloxacin (15.7%), tetracycline (12.9%), moxifloxacin (2.9%), erythromycin (2.9%), and telithromycin (2.9%) was observed. In species-level analyses, *U. urealyticum* showed higher rates of resistance to levofloxacin (20%), tetracycline (20%), and moxifloxacin (6.7%) than *U. parvum*, whereas no resistance to erythromycin or telithromycin was observed. In contrast, *U. parvum* exhibited resistance to erythromycin and telithromycin at the same rate (4.1%). Among the six samples with co-detection of *U. urealyticum* and *U. parvum*, resistance to levofloxacin and tetracycline was observed in five (83.3%) samples, while no resistance to moxifloxacin, erythromycin, or telithromycin was detected.

Among the 46 female patients with exclusive growth of *U. parvum*, levofloxacin resistance was detected in seven isolates (ages 20, 32, 38, 43, 64, 76, and 78 years), erythromycin resistance in two isolates (ages 10 and 48 years), telithromycin resistance in two isolates (ages 10 and 48 years), tetracycline resistance in one isolate (age 48 years), and moxifloxacin resistance in one isolate (age 32 years). Among the 10 female patients with exclusive growth of *U. urealyticum*, levofloxacin resistance was detected in three isolates (ages 28, 32, and 49 years), tetracycline resistance in three isolates (ages 28, 32, and 43 years), and moxifloxacin resistance in one isolate (age 49 years). Among the six female patients with co-growth of *U. parvum* and *U. urealyticum*, tetracycline resistance was identified in five isolates (ages 18, 23, 24, 28, and 50 years), and levofloxacin resistance was identified in one isolate (age 24 years). No antimicrobial resistance was detected among isolates recovered from the 13 male patients with *Ureaplasma* spp. growth.

Among the 12 female patients with *M. hominis* growth, clindamycin resistance was detected in one isolate (age 48 years), and no resistance to the other tested antibiotics was observed. No antimicrobial resistance was detected in the single isolate recovered from a male patient with *M. hominis* growth.

*Mycoplasma hominis* growth was detected by MYCOPLASMA IST3 in 13 samples, and antimicrobial susceptibility results were obtained. No resistance to levofloxacin, moxifloxacin, or tetracycline was observed, whereas clindamycin resistance was observed in 7.70% of isolates. The MIC (µg/mL) distributions and the corresponding susceptibility categories interpreted according to the CLSI M43-A guideline are presented in Table 6.

In our study, *Ureaplasma* spp. and *M. hominis* showed concomitant growth in 13 samples. In these samples, resistance to levofloxacin (23.08%), moxifloxacin (7.69%), erythromycin (7.69%), and telithromycin (7.69%) was observed more frequently than in samples with *Ureaplasma* spp. growth alone (Table 7).

## 3. Discussion

Genital mycoplasmas can cause a range of infections, primarily involving the urogenital tract. To determine whether they represent true etiologic agents and to guide treatment decisions, their presence should be demonstrated in appropriately collected clinical specimens, and AST should be performed. Clinical microbiology laboratories should therefore implement suitable diagnostic methods for these organisms and be able to perform AST when indicated.

In the presence of clinical symptoms, positivity rates, organism distribution, and test performance may vary depending on the specimen type (e.g., urine, urethral discharge, or genital swabs). The present study was conducted on midstream urine samples submitted with a presumptive diagnosis of urinary tract infection and analyzed using q-PCR and the MYCOPLASMA IST3 assay. Strauss et al. investigated *U. parvum* and *U. urealyticum* in urine samples using a multiplex PCR approach. In a study including first-void urine and endocervical, urethral, and vaginal swabs, Marovt et al. used PCR for species-level identification of culture-positive mycoplasmas [17,18].

In our study, *M. hominis* growth was detected by the MYCOPLASMA IST3 assay in 13 of the 75 evaluated patients; only one of these patients was male, while the remaining 12 were female. In a PCR-based study by Nassar et al. investigating the presence of *C. trachomatis*, *M. hominis*, *M. genitalium*, and *U. urealyticum* in patients with sterile pyuria, *M. hominis* was detected only in female patients. The authors reported that PCR identified a substantial number of *C. trachomatis*, *Mycoplasma* and *Ureaplasma* infections and recommended the use of PCR methods to detect these microorganisms in patients with sterile pyuria [7].

Min Young Lee et al. investigated the prevalence of *M. hominis* and *U. urealyticum*, their effects on pregnancy outcomes, and antimicrobial susceptibility patterns. Using vaginal swab specimens, they applied the Mycoplasma IST-2 assay (bioMérieux, Marcy-l’Étoile, France) for identification and susceptibility testing of *U. urealyticum* and *M. hominis*. They reported that among culture-positive samples, 82.7% yielded *U. urealyticum*, 0.3% yielded *M. hominis*, and 17% yielded concomitant growth of both organisms and noted an increasing trend in mixed *U. urealyticum*/*M. hominis* infections. In our study, concomitant growth of *Ureaplasma* spp. and *M. hominis* was detected in 13 samples; in these samples, the rates of resistance of *Ureaplasma* spp. to levofloxacin (23.08%), moxifloxacin (7.69%), erythromycin (7.69%), and telithromycin (7.69%) were higher than those observed in samples with *Ureaplasma* spp. growth alone. Lee et al. also reported josamycin susceptibility rates of 100% for *M. hominis* and 97.9% for *U. urealyticum*, whereas mixed isolates showed a markedly lower josamycin susceptibility rate (49.2%), emphasizing that mixed infections generally exhibit higher resistance levels than single-organism infections. They emphasized that *M. hominis* and/or *U. urealyticum* infections are highly prevalent in pregnant women, that resistance to antimicrobial agents appears to be increasing, and that these infections are associated with adverse pregnancy outcomes such as preterm birth. Accordingly, they highlighted the importance of timely identification of the causative organisms and prompt initiation of appropriate antibiotic therapy to support a safe pregnancy. They also recommended that organism identification and AST be implemented routinely in clinical laboratories [4].

Valentine-King et al., in a study of college-aged women experiencing a first episode of UTI, determined MIC values for 73 *Ureaplasma* spp. isolates (60 *U. parvum* and 13 *U. urealyticum*) and 10 *M. hominis* isolates and reported overall low resistance rates. In that study, which evaluated antimicrobial resistance only among urine isolates, all *M. hominis* and *U. urealyticum* isolates were susceptible, whereas two *U. parvum* isolates were resistant. One isolate was resistant to levofloxacin (MIC, 4 µg/mL) and the other to tetracycline (MIC, 8 µg/mL). To investigate the genetic mechanisms underlying resistance, the authors performed PCR-based analyses and identified tet (M) in the tetracycline-resistant isolate and an S83W mutation in parC in the fluoroquinolone-resistant isolate. When comparing MIC levels across *Ureaplasma* species, they found that *U. urealyticum* exhibited significantly higher MIC values for all tested antibiotics except doxycycline [3].

In our study, levofloxacin resistance (MIC ≥ 4 µg/mL) was detected in seven *U. parvum* samples, three *U. urealyticum* samples, and one sample with co-detection of *U. urealyticum* and *U. parvum*. Tetracycline resistance (MIC ≥ 2 µg/mL) was identified in one *U. parvum* sample, three *U. urealyticum* samples, and five samples with co-detection of *U. urealyticum* and *U. parvum*. Among the samples in which *M. hominis* was detected, no resistance to levofloxacin, moxifloxacin, or tetracycline was observed, whereas clindamycin resistance (MIC ≥ 0.5 µg/mL) was detected in one sample.

Commercial culture-based assays and molecular methods are increasingly used for the diagnosis of genital mycoplasmas. Published studies have reported their prevalence and demonstrated that detection rates may vary depending on the diagnostic approach applied. The advantages of PCR include rapid turnaround time, high analytical sensitivity, and the ability to provide an approximate indication of bacterial burden. However, PCR-based assays are limited to the targets included in the panel; moreover, the development of panels incorporating antimicrobial resistance profiles is challenging, and the presence of resistance genes does not always correlate with phenotypic resistance. In our study, the MYCOPLASMA IST3 assay was easier to implement and provided results more rapidly than conventional culture, thereby partially overcoming key limitations of standard culture methods. Nevertheless, despite a shorter overall workflow, biochemical commercial culture-based tests still require 24–48 h of incubation, which represents a disadvantage compared with PCR assays that can yield results within a few hours. Although PCR may offer semi-quantitative information on bacterial load, culture-based methods remain necessary when quantitative assessment and phenotypic susceptibility profiling are required [3,10,14].

The antibiotic concentrations included in the MYCOPLASMA IST3 assay used for AST in our study are consistent with CLSI breakpoints, and the assay is straightforward to perform and interpret. In an international multicenter study by Ian Boostrom et al., the performance of the MYCOPLASMA IST3 assay was evaluated and reported to be highly sensitive and specific for the identification of *M. hominis* and *Ureaplasma* spp.; importantly, in mixed infections, AST results for both organisms could be accurately interpreted independently. In our study, concomitant growth of *Ureaplasma* spp. and *M. hominis* was detected in 13 samples, and both identification and AST results were readily interpretable. In these 13 co-growth samples, rates of resistance to levofloxacin (23.08%), moxifloxacin (7.69%), erythromycin (7.69%), and telithromycin (7.69%) were higher than those observed in samples with *Ureaplasma* spp. growth alone [10].

In many laboratories, AST for *Mycoplasma* and *Ureaplasma* species is performed infrequently. Nevertheless, increasing rates of resistance to commonly used agents, including tetracyclines, fluoroquinolones, macrolides, and clindamycin, have been reported. Resistance to levofloxacin was observed in 15.71% of isolates; to tetracycline in 12.9%; and to moxifloxacin, erythromycin, and telithromycin in 2.9% each. Clindamycin resistance was observed in 7.7% of *M. hominis* isolates. These findings highlight the importance of performing AST and monitoring local resistance patterns to support effective therapy [19,20,21].

*Ureaplasma* spp. and *M. hominis* are common colonizers of the lower urogenital tract, and their detection does not always indicate infection. In the clinical interpretation of suspected infection, factors such as organism burden, the presence of compatible symptoms, and markers of infection (e.g., pyuria) may be helpful. In this study, urine samples were submitted with a presumptive diagnosis of UTI; however, clinical data—including symptoms and related findings—were not available. Therefore, our results should be interpreted not as confirmation of etiologic infection in all cases but, rather, as findings describing the yield of MYCOPLASMA IST3 growth detection and the antimicrobial susceptibility profile among *Ureaplasma* spp. q-PCR-positive urine samples [18,22,23].

## 4. Materials and Methods

**Figure 5:**
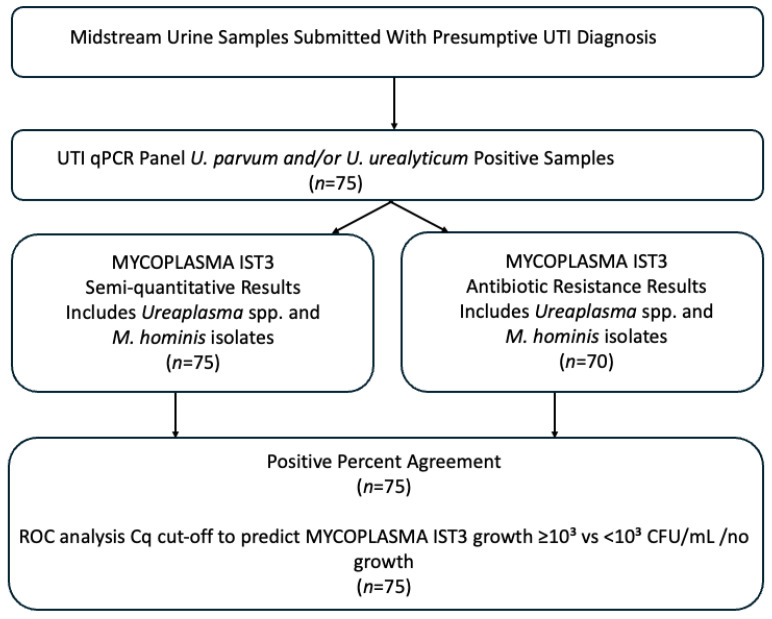
Study workflow.

In this study, midstream urine samples submitted to our laboratory with a presumptive diagnosis of urinary tract infection were tested by q-PCR without delay. A total of 75 samples in which *U. parvum* and/or *U. urealyticum* were detected were analyzed on the same day using the MYCOPLASMA IST3 assay (bioMérieux, Marcy-l’Étoile, France). Repeat samples from the same patient were excluded from the study. Demographic variables (age and sex) and clinical information (referring clinic and presumptive diagnosis) were obtained from the hospital laboratory information system accompanying the test requests (Figure 5).

The urinary tract infections q-PCR panel (Bioeksen, İstanbul, Türkiye) is a 29-target multiplex assay that includes *U. parvum* and *U. urealyticum*; however, *M. hominis* is not among its targets. According to the manufacturer, the assay’s analytical sensitivity is 100–500 genome copies/mL, with a reported limit of detection of 125 genome copies/mL for *U. urealyticum*/*U. parvum* and an analytical specificity of 100%. Because the q-PCR panel used in this study was not designed to assess antimicrobial susceptibility, we did not perform susceptibility testing using this method.

### 4.1. q-PCR Assay

For q-PCR analysis, 200 µL of each urine sample was processed using rapid nucleic acid extraction (Bioeksen^®^, Türkiye) on a Zybio EXM 3000 instrument (Zybio Inc., Chongqing, China) with a magnetic bead-based extraction protocol. Subsequently, 10 µL of eluate was transferred from the final well of the extraction cartridge to the urinary tract infections PCR panel strips (Bioeksen, Türkiye). The strips were loaded onto a Bio-Rad CFX96 Touch™ PCR system (Bio-Rad Laboratories, Hercules, CA, USA), and amplification was performed according to the manufacturer’s instructions.

Results were interpreted only after confirming that the positive control, negative control, and internal controls performed as expected.

### 4.2. MYCOPLASMA IST3 Assay

The MYCOPLASMA IST3 assay is a liquid culture-based in vitro diagnostic test developed for the detection, identification, and antimicrobial susceptibility testing of genital mycoplasmas. The reported sensitivity and specificity are 98.4% and 99.7%, respectively, for *Ureaplasma* spp. and 95.7% and 100%, respectively, for *M. hominis* [10]. In the MYCOPLASMA IST3 assay, the metabolism of urea and/or arginine by the microorganisms increases the pH, resulting in a red color change of the medium; growth is assessed based on this color change.

The MYCOPLASMA IST3 strips provide semi-quantitative growth results for *Ureaplasma* spp. at ≥10^3^, ≥10^4^, and ≥10^6^ CFU/mL and for *M. hominis* at ≥10^4^ and ≥10^6^ CFU/mL. AST is based on MIC readouts using predefined antibiotic concentrations. For *Ureaplasma* spp., five agents are included: levofloxacin (2 and 4 mg/L), moxifloxacin (2 and 4 mg/L), tetracycline (1 and 2 mg/L), erythromycin (8 and 16 mg/L), and telithromycin (4 mg/L). For *M. hominis*, four agents are included: levofloxacin (1 and 2 mg/L), moxifloxacin (0.25 and 0.5 mg/L), tetracycline (4 and 8 mg/L), and clindamycin (0.25 and 0.5 mg/L).

After mixing 200 µL of urine with the Mycoplasma R1 solution, 3 mL of the mixture was transferred onto the lyophilized Mycoplasma R2 pellet. From the homogenized suspension (R1 + R2 + urine), 55 µL was dispensed into each of the 25 wells of the MYCOPLASMA IST3 strip. Two drops of mineral oil were added to each well. The strip was sealed, and both the MYCOPLASMA IST3 strip and the Mycoplasma R2 vial were incubated under aerobic conditions at 36 ± 2 °C for 48 h.

At 24 and 48 h, color changes in the Mycoplasma R2 vial and the strip wells were interpreted as follows: yellow indicated no growth, whereas an orange-to-red color indicated growth. Semi-quantitative results and MIC interpretations for the tested antibiotics were evaluated according to the CLSI M43-A guideline [16].

### 4.3. Statistical Analysis

Data were analyzed using SPSS v25.0. Categorical variables are presented -s *n* (%), and normality was assessed using the Shapiro–Wilk test. Because only q-PCR positive *Ureaplasma* spp. samples were included, methodological comparison was based on the positive percent agreement (PPA) rather than sensitivity/specificity; 95% confidence intervals were calculated using the Wilson method. Samples with no growth in the MYCOPLASMA IST3 assay were reported as discordant results.

Receiver operating characteristic (ROC) analysis was performed to determine the optimal PCR-derived Cq cutoff for prediction of *Ureaplasma* spp. growth in the MYCOPLASMA IST3 assay. All 75 q-PCR-positive samples were included, and the binary outcome was defined as MYCOPLASMA IST3 growth at ≥10^3^ CFU/mL versus < 10^3^ CFU/mL/no growth. The cutoff point was defined as the threshold providing the highest sensitivity and specificity. The area under the curve (AUC) was interpreted as follows: values between 1.00–0.90 were considered excellent, 0.90–0.80 good, 0.80–0.70 fair, 0.70–0.60 poor, and 0.60–0.50 indicative of very poor discriminatory ability. For all hypothesis tests, the Type I error rate was set at α = 0.05.

All included samples had complete q-PCR results and MYCOPLASMA IST3 growth readouts; therefore, no imputation was performed, and analyses were conducted using complete-case data. Variables that were not available (e.g., symptoms, pyuria, and treatment response) were not included in the analyses.

Five q-PCR-positive samples showed no growth in MYCOPLASMA IST3 and were classified as discordant. These samples were not excluded from agreement analyses and were included in the denominator for positive percent agreement (PPA) as ‘no growth’. Because MIC/susceptibility results were generated only for growth-positive isolates, these five samples were excluded from MIC distribution and resistance-rate analyses by design.

## 5. Strengths and Limitations

This study was conducted using clinical specimens and reflects routine laboratory practice. The microbiological evaluation assessed a diagnostic approach integrating both molecular and phenotypic methods. Given the growing concern regarding antimicrobial resistance, our findings are also valuable in providing regional resistance rates and data that may inform empirical treatment strategies.

We aimed to evaluate the feasibility, performance characteristics, inter-method agreement, and positivity rates of two diagnostic approaches; however, our methodological comparison was limited by several factors. First, the q-PCR urinary panel used in this study targets *U. parvum* and *U. urealyticum* but does not include *M. hominis*; therefore, we could not assess inter-method agreement or diagnostic performance for *M. hominis*, and conclusions regarding the MYCOPLASMA IST3 assay for this organism are limited to culture-based detection and susceptibility findings. Second, the MYCOPLASMA IST3 assay reports *Ureaplasma* at the genus level and does not differentiate *U. parvum* from *U. urealyticum*; accordingly, species-level interpretation of phenotypic resistance patterns should be made cautiously, particularly in samples with co-detection.

A primary limitation is selection bias due to the restriction of inclusion to q-PCR-positive *Ureaplasma* spp. samples. This sampling frame limits the generalizability of our findings to unselected patients undergoing routine evaluation for suspected urinary tract infection, including PCR-negative specimens. Classical diagnostic accuracy metrics such as sensitivity, specificity, and negative predictive value cannot be estimated from our dataset. Therefore, the positive percent agreement reported here should be interpreted as agreement conditional on q-PCR positivity, i.e., the proportion of q-PCR-positive samples that yielded growth in the MYCOPLASMA IST3 assay, and should not be construed as the sensitivity of either method in a general clinical population.

Because ROC modeling was restricted to q-PCR-positive samples, the resulting cutoff is intended for use within q-PCR-positive patients and is not generalizable to the screening of unselected populations. In addition, the q-PCR Cq cutoff and the corresponding AUC were derived and evaluated in the same dataset (*n* = 75), which may lead to optimistic performance estimates. At the selected Cq cutoff, sensitivity (73.4%) and specificity (66.7%) were modest, indicating limited discriminative utility; therefore, the threshold should be used as an adjunct to—rather than a substitute for—microbiological and clinical interpretation. Our ROC outcome was defined as predicting MYCOPLASMA IST3 growth at ≥10^3^ CFU/mL (a higher-burden phenotype), which may reasonably correspond to lower Cq values. Because Cq values and optimal thresholds are assay- and platform-dependent, the proposed cutoff should be interpreted in the context of our specific q-PCR assay and study design, and it requires validation before being generalized to other assays or patient populations.

Finally, independent clinical outcome data (e.g., symptoms, pyuria, and treatment response) were not available. Therefore, we were unable to assess the predictive value of MYCOPLASMA IST3 MIC/susceptibility results for treatment response or clinical cure, and any clinical inference based on these findings should be interpreted with caution.

## 6. Conclusions

In our study, among the 75 q-PCR-positive *Ureaplasma* spp. samples, *U. parvum* was detected in 54 (72%), while *U. urealyticum* was detected in 15 (20%), with co-detection of *U. urealyticum* and *U. parvum* in 6 (8%). Growth was detected by the MYCOPLASMA IST3 assay in 70/75 samples (positive percent agreement, 93.33%), whereas no growth was observed in 5/75 (discordance, 6.66%). Based on MYCOPLASMA IST3 AST results, among the 70 samples with *Ureaplasma* spp. growth, resistance to levofloxacin was observed in 15.71%; to tetracycline in 12.9%; and to moxifloxacin, erythromycin, and telithromycin in 2.9% each. Among the 13 samples with *M. hominis* growth, no resistance to levofloxacin, moxifloxacin, or tetracycline was observed, whereas clindamycin resistance was observed in 7.7%. These susceptibility findings indicate that regional resistance patterns should be considered when selecting therapy. Laboratories relying solely on conventional diagnostic methods should evaluate their patient populations, available resources, and the characteristics of the target organisms and testing platforms and implement appropriate diagnostic strategies—such as PCR and/or commercial culture-based assays—to detect these pathogens and assess antimicrobial resistance, thereby providing clinically relevant guidance for treatment.

## Figures and Tables

**Table 1:** Distribution of patients by clinical department.

Clinic	*n*	%
Obstetrics and Gynecology	33	44
Urology	21	28
Internal Medicine	20	26.67
Pediatrics	1	1.33
Total	75	100

**Table 2:** Distribution of presumptive clinical diagnoses.

Presumptive Clinical Diagnosis	*n*	%
Urinary tract infection	65	86.67
Vaginitis	7	9.33
Prostatic hypertrophy/prostatitis	3	4
Total	75	100

**Table 3:** MYCOPLASMA IST3 growth results (CFU/mL) in PCR-positive *U. parvum* and *U. urealyticum* samples.

3A. *Ureaplasma* spp. Results							
	PCR Results	IST 3 Results					
*Ureaplasma* spp.	*Ureaplasma* spp.	<10^3^	≥10^3^	≥10^4^	≥10^6^	No Growth	Total
	*U. parvum*	3	3	27	16	5	54
	*U. urealyticum*	3	3	6	3	0	15
	*U. parvum+* *U. urealyticum*	0	1	1	4	0	6
	Total	6	7	34	23	5	75
**3B. *M. hominis* Results**							
		IST 3 Results					
*M. hominis*		<10^4^		≥10^4^	≥10^6^	No Growth	Total
		4		5	4	62	75

**Table 4:** ROC analysis results for Cq values in samples with ≥10^3^ CFU/mL *Ureaplasma* spp. growth.

IST3 Growth	AUC (%95)	Cutoff	*p*	Sensitivity (%)	Specificity (%)
≥10^3^	0.891 (0.785–0.996)	<20.17	<0.001	73.40%	66.70%

**Table 5:** Distribution of MIC (µg/mL) results and susceptibility categories for *Ureaplasma* spp.

			*U. urealyticum* *+* *U. parvum*		*U. urealyticum*		*U. parvum*		Total	
Antibiotics	MIC (μg/mL)	Category	*n*	%	*n*	%	*n*	%	*n*	%
Levofloxacin	≤2	S	5	83.3	12	80	42	85.7	59	84.3
	≥4	R	1	16.7	3	20	7	14.3	11	15.7
Moxifloxacin	≤2	S	6	100	14	93.3	48	98	68	97.1
	≥4	R	0	0	1	6.7	1	2	2	2.9
Tetracycline	≤1	S	1	16.7	12	80	48	98	61	87.1
	≥2	R	5	83.3	3	20	1	2	9	12.9
Erythromycin	≤8	S	6	100	15	100	47	95.9	68	97.1
	≥16	R	0	0	0	0	2	4.1	2	2.9
Telithromycin	≤4	S	6	100	15	100	47	95.9	68	97.1
	>4	R	0	0	0	0	2	4.1	2	2.9

S = Susceptible; R = Resistant. The MYCOPLASMA IST3 assay uses a strip-based, predefined concentration format rather than providing an exact MIC for each isolate. Susceptibility categorization was derived from growth/no growth in the strip concentration steps according to CLSI breakpoints. CLSI breakpoints (µg/mL): levofloxacin, S ≤ 2 and R ≥ 4; moxifloxacin, S ≤ 2 and R ≥ 4; tetracycline, S ≤ 1 and R ≥ 2; erythromycin, S ≤ 8 and R ≥ 16; telithromycin, S ≤ 4 [16].

**Table 6:** Distribution of MIC (µg/mL) results and susceptibility categories for *M. hominis*.

Antibiotics	MIC (μg/mL)	Category	*n*	%
Levofloxacin	≤1	S	13	100
	≥2	R	0	0
Moxifloxacin	≤0.25	S	13	100
	≥0.5	R	0	0
Tetracycline	≤4	S	13	100
	≥8	R	0	0
Clindamycin	≤0.25	S	12	92.3
	≥0.5	R	1	7.7

S = Susceptible; R = Resistant. The MYCOPLASMA IST3 assay uses a strip-based, predefined concentration format rather than providing an exact MIC for each isolate. Susceptibility categorization was derived from growth/no growth in the strip concentration steps according to CLSI breakpoints. CLSI breakpoints (μg/mL); levofloxacin, S ≤ 1 and R ≥ 2; moxifloxacin, S ≤ 0.25 and R ≥ 0.5; tetracycline, S ≤ 4 and R ≥ 8; clindamycin S ≤ 0.25 and R ≥ 0.5 [16].

**Table 7:** Distribution of MIC (µg/mL) results and susceptibility categories for *Ureaplasma* spp. in samples with concomitant growth of *Ureaplasma* spp. and *M. hominis*.

			*Ureaplasma* spp. + *M. hominis*		*Ureaplasma* spp.	
Antibiotics	MIC (μg/mL)	Category	*n*	%	*n*	%
Levofloxacin	≤2	S	10	76.92	49	86
	≥4	R	3	23.08	8	14
Moxifloxacin	≤2	S	12	92.31	56	98.2
	≥4	R	1	7.69	1	1.8
Tetracycline	≤1	S	13	100	52	91.2
	≥2	R	0	0	5	8.8
Erythromycin	≤8	S	12	92.31	56	98.2
	≥16	R	1	7.69	1	1.8
Telithromycin	≤4	S	12	92.31	56	98.2
	>4	-	1	7.69	1	1.8

S = Susceptible; R = Resistant.

## Data Availability

The data presented in this study are available on request from the corresponding author due to privacy/ethical restrictions.
